# Chiral Separations in Preparative Scale: A Medicinal Chemistry Point of View

**DOI:** 10.3390/molecules25081931

**Published:** 2020-04-21

**Authors:** Madalena M.M. Pinto, Carla Fernandes, Maria E. Tiritan

**Affiliations:** 1 Laboratório de Química Orgânica e Farmacêutica, Departamento de Ciências Químicas, Faculdade de Farmácia da Universidade do Porto, 4050-313 Porto, Portugal; cfernandes@ff.up.pt (C.F.); elizabeth.tiritan@iucs.cespu.pt (M.E.T.); 2 Centro Interdisciplinar de Investigação Marinha e Ambiental (CIIMAR), Edifício do Terminal de Cruzeiros do Porto de Leixões, 4050-208 Matosinhos, Portugal; 3 CESPU, Instituto de Investigação e Formação Avançada em Ciências e Tecnologias da Saúde (IINFACTS), 4585-116 Gandra PRD, Portugal

**Keywords:** chirality, chiral separations, preparative scale, liquid chromatography, supercritical fluid chromatography, counter-current chromatography, membranes, drug discovery, drug development

## Abstract

Enantiomeric separation is a key step in the development of a new chiral drug. Preparative liquid chromatography (LC) continues to be the technique of choice either during the drug discovery process, to achieve a few milligrams, or to a scale-up during the clinical trial, needing kilograms of material. However, in the last few years, instrumental and technical developments allowed an exponential increase of preparative enantioseparation using other techniques. Besides LC, supercritical fluid chromatography (SFC) and counter-current chromatography (CCC) have aroused interest for preparative chiral separation. This overview will highlight the importance to scale-up chiral separations in Medicinal Chemistry, especially in the early stages of the pipeline of drugs discovery and development. Few examples within different methodologies will be selected, emphasizing the trends in chiral preparative separation. The advantages and drawbacks will be critically discussed.

## 1. Introduction

Considering the broad scope of Medicinal Chemistry; “chemistry-based discipline, also involving aspects of biological, medical and pharmaceutical sciences; it is concerned with the invention, discovery, design, identification and preparation of biologically active compounds, the study of their metabolism, the interpretation of their mode of action at the molecular level and the construction of structure—activity relationships” [1]; it is clear that a large pleiad of compounds, belonging to, for instance, agro-chemicals and drugs—small molecules or biological products—can be embodied in this definition. Among these, chiral compounds play a special role taking in account the behavior of the enantiomeric pairs considering that separated isomers can display different biological/pharmacological activities [2,3]. In chiral drugs, knowledge about the stereochemistry is crucial and accompanies several steps of drug discovery and development. Thus, it can influence absorption, distribution, metabolism and excretion, as well as the molecular mechanism of action, through the phenomenon of chiral molecular recognition [2,4].

Chiral drugs embody 40–50% of the market, covering different therapeutic classes [5]. In the majority of the cases, enantiomerically pure drugs have been increasingly developed for the pharmaceutical market due to their undeniable benefits, such as superior potency and safety, comparing with racemates [6]. This preference is obvious and, in 2015, from the ten most prescribed pharmaceuticals in the United States, eight were chiral and, among them, seven were commercialized as single enantiomers [7]. In 2018, nine of the ten best selling drugs were chiral [8]. Among them, seven were complex molecules comprising intrinsic chirality, including recombinant fusion proteins; the other two were small molecules being one of them commercialized as single enantiomer. 2019 followed the same tendency of 2018 and the projection until the year 2024 pointed Lipitor^®^ as second top-selling drugs [9]. Besides, there are many chiral drugs market as single enantiomers that were previously available as racemates (chiral switches), enhancing a new trend not only for public health consideration but also for economic purposes [5,10].

**Figure 1:**
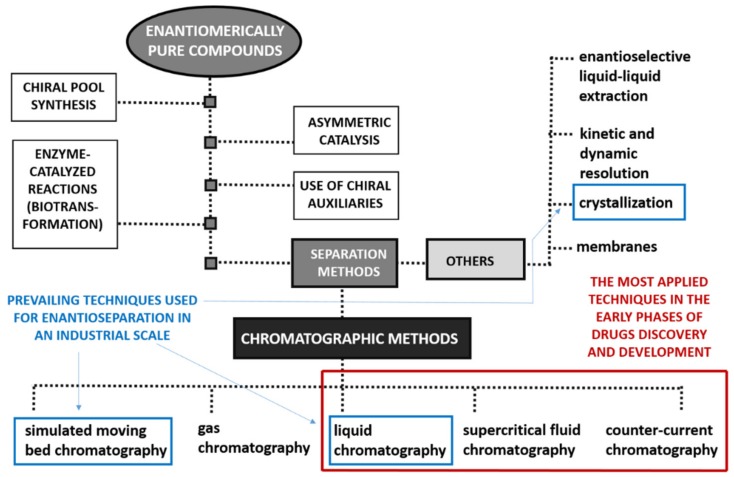
Different ways to obtain enantiomerically pure compounds.

In response to the exponential research in Medicinal Chemistry concerning the invention, discovery, design, identification and preparation of new biologically active compounds in enantiomerically pure form, aligned to the stricter requirements from regulatory authorities to patent new chiral drug [11], the development of methods for enantioselective production of chiral compounds as well as evaluation of their enantiomeric purity, proved to be essential. Accordingly, different enantioselective synthetic methodologies were developed [12,13] in addition to diverse analytical and preparative techniques [14,15]. Nevertheless, pure enantiomers can be achieved using several methods as illustrated in Figure 1.

At the early stage of drug discovery and development, a large library of molecules is required in milligrams amount for biological screening. In this stage, the enantioselective synthesis may not be efficient, insofar as it may require individual synthetic pathways to reach the enantiomers increasing time and cost of the process when compared to non-enantioselective synthesis. Furthermore, as is mandatory by the FDA’s policy statement both the enantiomers of the new drug candidate are needed for biological screening, consequently, the synthesis of the racemates and further enantioseparation to achieve both enantiomers is the better strategy [16,17]. At these stages, it is crucial to know, as early as possible, the difference in pharmacokinetic, pharmacodynamic and toxicological properties associated with each enantiomer to rapidly move on to the next step. Thus, preparative scale separation of enantiomers enables to accelerate drug discovery and development [18].

Over the last decades, several fundamental reviews and book chapters assembled the main achievements related to preparative separation of enantiomers [16,19,20,21,22,23,24]. In this overview, the currently most applied techniques and the recently progresses achieved on preparative chiral separations are brief reported, focusing in the importance to achieve pure enantiomers at the early phases of the pipeline of drugs discovery and development. Recent examples within different methodologies will be selected, emphasizing the chiral separation trends on preparative and semi-preparative scale.

## 2. Trends in Chiral Separations on Preparative Scale in Early Phases of Drug Discovery and Development

**Figure 2:**
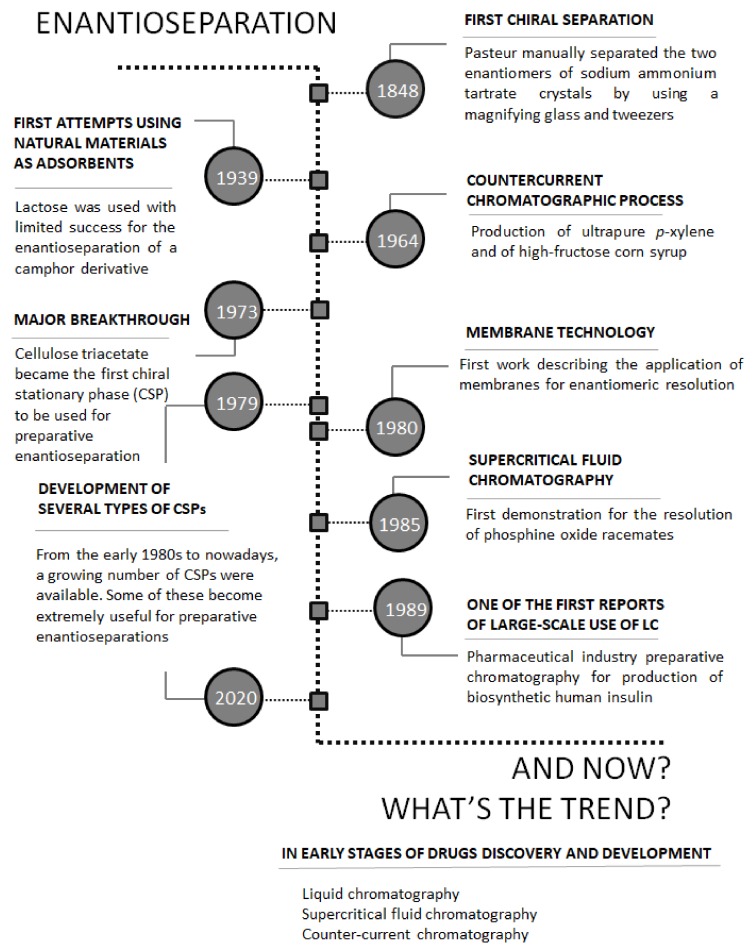
Timeline of the most important landmarks of enantioseparation.

As stated before, concerning the Medicinal Chemistry point of view, the separation of enantiomers in a preparative scale is undoubtedly a matter of crucial importance. In Figure 2, some of the most important landmarks of enantioseparation are summarized [17].

Over the years, the improvement of new separation techniques, the introduction of a large variety of materials used as chiral selectors, in addition to the instrumental and technical progress associated with preparative-scale separation of the enantiomers [25,26,27,28] have greatly contributed to the discovery and development process of chiral drugs. This trend occurred both in the early stage of drugs discovery, to achieve few milligrams of enantiomerically pure compounds, and in the stage of drug development to scale-up the quantities of enantiomers, to 100 g to 10 kg. For further development steps as well as in an industrial perspective (production scale), frequently only one enantiomer is needed being its amount considerably greater (5–100 kg to tons, respectively). In these stages, the costs and scale-up potential of the method become a very important issue to be considered [17]. There are prevailing resolution techniques and strategies to obtain the enantiomers in this scale including crystallization [29,30], simulated moving bed (SMB) [31], and liquid chromatography (LC) [14,22,27]. Leek H. et al. [32] recently reported a strategy for large-scale isolation of enantiomers in drug discovery, affording different options that involved different chromatographic techniques together with crystallization. It is also important to highlight that these techniques were also successfully applied for multi-gram preparation of enantiomers. Recent examples include the separation of stable enantio-enriched POCOP-based planar chiral pincer complexes by crystallization [33] and the enantioseparation of metalaxyl racemate by SMB chromatography [34]. Among the separation approaches, enantioselective LC is a very promising technique that has attracted great attention from the pharmaceutical industry for the production scale of enantiomerically pure drugs [35,36,37].

### 2.1. Liquid Chromatography

LC has a key role for medicinal chemists, being a crucial tool for the preparative enantioseparation in all stages of drug discovery and development [27,38] (Figure 1). Diverse technological options can be applied for the separation of enantiomers in a preparative scale, including classical batch column chromatography [39,40], recycling loop chromatography [41,42], VARICOL (multicolumn continuous chromatography, an improved version of SMB) technology [43,44], among others. LC has benefited from enormous development for the last four decades, generating vast technical experience and amount of scientific data reported in literature. In the theoretical point of view, the knowledge in physical chemistry of liquid/solid adsorption is almost ubiquitous. Moreover, the scale-up is straightforward, based on rules of thumbs such as scaling the volumetric flow and injection volume to the square of the ration of the column radius. Another advantage of LC compared with other techniques, such as supercritical fluid chromatography (SFC), is that instrumentation is simpler, less expensive and there is no need for electricity to cool or heat carbon dioxide, for example [26].

The increasing success of chromatographic methods is related to the improvement of chromatographic instrumentation as well as the development of versatile and highly efficient chiral stationary phases (CSPs) allowing the resolution of diverse chemical classes of enantiomers. Nevertheless, although the development of new CSPs is a continuous and challenger issue covering various types of CSPs [45,46], and considerable progress has been achieved to gain the insight of important principles of chiral recognition mechanisms [47,48,49], only few CSPs comprise suitable characteristics and are effective for preparative separation of enantiomers [27,50].

The main requirements of the CSPs for efficient enantioseparation in a preparative scale are based on adequate enantiorecognition ability together with a high-loading capability [38]. Additionally, ideal CSPs for preparative applications should be readily available and/or developed to be competitive in economic aspects, compared with other recognized enantioseparation techniques. If all these requirements are being met, the chromatographic preparative separation will afford the desired isolated enantiomers. Among the different types of CSPs, polysaccharide-based are the most used due to their versatility, high-loading capability, and enantiodiscrimination performance [36,40,41,51,52]. Despite the solvent limitation in mobile phase composition, the polysaccharide-based CSPs are compatible with normal, reversed, polar organic and polar ionic elution modes. Moreover, immobilized polysaccharides emerged as a reliable alternative allowing the use of a broader selection of solvents as mobile phases [53].

**Figure 3:**
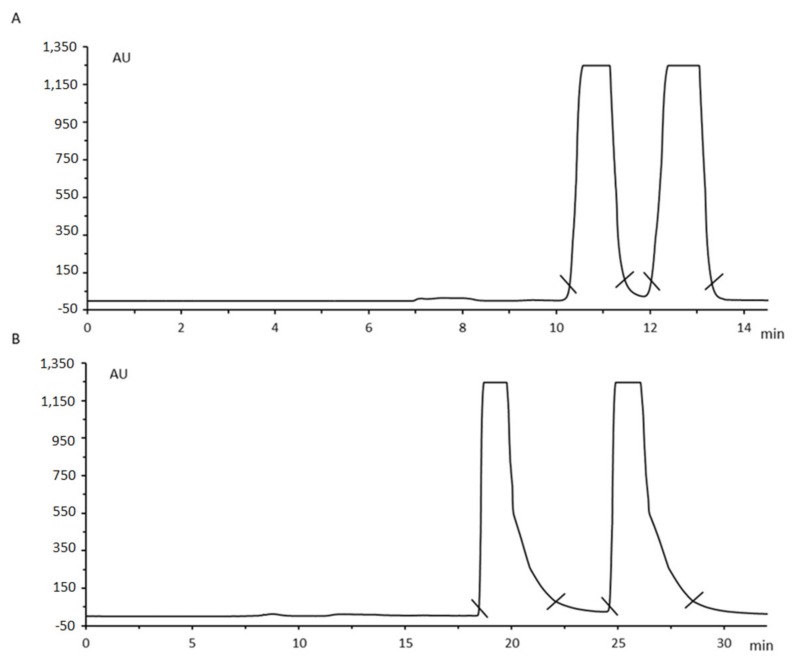
Liquid chromatography enantioseparation chromatogram of pentedrone (**A**) and methylone (**B**). Chromatographic conditions: Chiralpak^®^ AS-H column, mobile phase Hex/2-PrOH (97:3, *v*/*v*) for A and Hex/2-PrOH (85:15, *v*/*v*) for B, flow rate 2 mL/min, UV detection 254 nm (reprint permission from [61]).

Recent examples of applications at preparative scale on polysaccharide-based CSPs include: the enantioseparation of tetrahydro-α-(1-methylethyl)-2-oxo-1(2*H*)-pyrimidineacetic acid (TPA), a key intermediate for the synthesis of HIV protease inhibitors (enantiomeric purity of 99%) [54]; racemic coumarin derivatives [55]; isoborneol enantiomers [56]; high-affinity 5-HT7 receptor ligand (enantiomeric purity of 100%) [57]; synthetic E2F inhibitor HLM006474 (enantiomeric ratio higher than 99.5/0.5) [58]; novel pan-sigma receptor (SR) modulators, potentially useful in cancer treatment (enantiomeric excess higher than 95%) [59]; among others. Recently, we reported the enantioseparation of cathinone derivatives (amphetamine-like stimulant effects) at multi-milligram scale using the same type of CSPs (Figure 3) [60,61]. The excellent enantioresolution obtained allowed the collection of highly pure enantiomeric fractions achieving, for example, enantiomeric ratios higher than 98% for both methylone and pentedrone enantiomers [61].

Synthetic polymers, such as polyacrylamide and the cross-linked di-allyltartardiamide, as well as brush-type CSPs (including neutral and anion exchange small molecules as chiral selectors), followed by cyclodextrin and macrocyclic antibiotic-based CSPs, are also frequently used for preparative enantioseparation purposes [27].

In addition to the CSP, the mobile phase also has an outstanding role in the overall chromatographic process and its consumption should be optimized [62]. The solubility and stability of the compounds in the mobile phase is important to achieve a good productivity of the separation. Besides, a mobile phase that is easy to remove, less expensive and more environmentally is always desirable. From a green chemistry point of view, the use of a large amount of organic solvents as mobile phase in LC has negative impact on the environment, personal health and safety risk [63,64]. However, normal and polar organic elution modes are preferable to the reversed elution mode because they are easier to be removed. Greener alternatives are a trend consistent with the increasing popularity of pure polar organic mobile phases as ethanol in LC and the reduced use or absence of organic solvents as preconized by SFC [65]. Higher efficiency and loadability, to increase the productivity of preparative enantioseparation, as well as faster flow-rate and column equilibration, leading to shorter analysis and cycle times without loss of resolution, are also desirable [26].

### 2.2. Supercritical Fluid Chromatography

SFC has been a field of great interest since the first report of a chiral separation using this technique by Mourier et al. [66], in 1985. Since then, and particularly in the last few years, this rapidly emerging technique is gaining popularity and proved to be a powerful enantioselective chromatographic technique for both analytical and preparative applications [26,67,68,69]. There are several examples of pharmaceutical separations by SFC in preparative-scale [70]. The use of carbon dioxide as a mobile phase in SFC offers many benefits including high flow rate, short equilibration time and reduced solvent consumption resulting in limited environmental impact. Environmental factor (E Factor) was calculated for four column sizes for sequence and stacked modes of injection using Chiralpak^®^ AD-H amylose *tris*(3,5-dimethylphenylcarbamate) polysaccharide-based stationary phase columns of various dimensions for the purification of propranolol. Production rate (mg/h), productivity (kilograms of racemate separated per kilogram of CSP per day; kkd) and solvent usage (L/g) were evaluated. A semi-preparative column (30 mm i.d.) yielded the best production rate of 467 mg purified per hour but had the worst environmental impact with an E factor of approximately 56,414 due to the solvent volume used during column equilibration. Considering the cost-effectiveness of the process, production rates increased with column dimensions. Indeed, a larger quantity of racemate could be purified per hour on a larger column (from 10.9 to 467 mg/h). In contrast, the highest kkd productivity (0.176 in stacked injection mode) was obtained on the smallest column (150 × 4.6 mm i.d.) because of the lower quantity of stationary phase [65]. Regarding E factors for three columns, they were similar, between 15,191 and 16,293; however, for the fourth and largest column, the E factor was 24,269 (without considering the equilibration step). Thus, the authors concluded that, if the goal is reduced environmental impact, the smallest column should be used. Despite the high E factor values, preparative SFC can be considered a greener process when compared to LC because a large proportion of the mobile phase comprises CO_2_ (a greenhouse gas) collected as by-product from industrial process [65].

Other attributes of this technology are fast method development cycle, feasibility in scale-up, high throughput, fast analysis time, wide polarity compatibility, low cost of the mobile phase and high-column efficiency [26,68]. These advantages of SFC and the further developments in instrumentation, as well as availability of a wide range of CSPs, made this technique become a strong competitor to LC for both analytical and preparative scale separation [68,69]. Moreover, in many cases, would make it a better choice than LC for preparative purification of chiral compounds in drug discovery, where time constraints are crucial [68,71].

**Figure 4:**
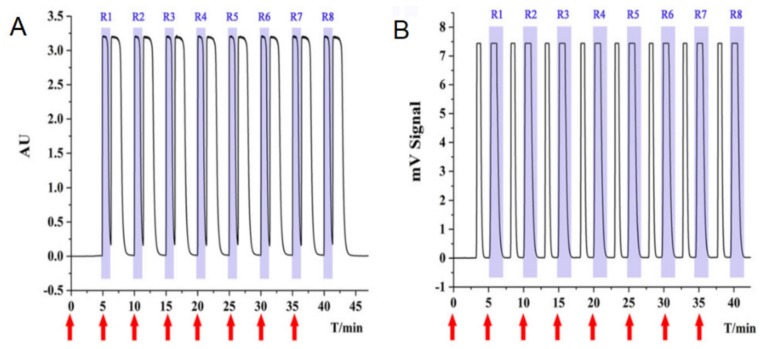
Preparative chromatograms of SKF 93,505 in: (**A**) Liquid chromatography under polar organic mode on Chiralcel OJ column; and in (**B**) Supercritical Fluid Chromatography on Chiralpak AS column. Chromatographic conditions in (**A**): Mobile phase, 100% MeOH; flow rate, 20 mL/min. Chromatographic conditions in (**B**): Mobile phase, CO_2_/MeOH (55:45, *v*/*v*); flow rate, 60 g/min; back pressure: 17.0MPa. Column dimensions (250 × 20 mm, i.d., 10 μm); sample concentration, 15 mg/mL; each injection volume, 2 mL (reprint permission from [74]).

Some recent works emphasized the advantages of this technique compared to the LC [72,73,74]. For example, Wu, D.R. et al. [73] reported an SFC preparative enantioseparation of acid and basic racemates using two polysaccharide-derived CSPs and methanol as co-solvent without trifluoroacetic acid (TFA) or diethylamine (DEA), respectively, as additives. The use of methanol without acid or basic additives proved to be a benefit of SFC over conventional chiral LC, eliminating the risk of potential degradation of racemates and providing a higher throughput. Cheng, L. et al. [74] reported an efficient SFC preparative separation of a key synthetic intermediate for the cardiotonic agent levosimendan (SFK 93505) using also a polysaccharide-derived CSP and CO_2_/methanol without additive as mobile phase. The preparative enantioseparation of SFK 93,505 was also successfully performed by LC on a polysaccharide-based CSP in polar organic mode (Figure 4). By comparison of the enantioseparation efficiencies, it was found that SFC exhibited a better productivity (98%) with high enantiomeric excess values (100%).

Similarly to LC, for the majority of the chiral SFC separations, the polysaccharide-based CSPs are the most used, in addition to Pirkle-type, cyclodextrin-based and macrocyclic antibiotic-based CSPs [75]. Recent SFC preparative separation of enantiomers embrace the enantioseparation of 3-carboxamido-5-aryl isoxazole derivatives on amylose- and cellulose- based CSPs (enantiomeric purity higher than 98%) [76]. Using the same type of CSPs, a preparative-scale SFC separation of highly potent and selective Bruton’s tyrosine kinase inhibitor atropisomers were successfully developed [77]. Adequate amounts of atropisomers were obtained, with high enantiomeric purity (99.8%) to support further toxicology studies. Another recent example, reports the separation of stereoisomers of 7-oxa-bicyclo[2.2.1]heptene sulfonate, a selective estrogen receptor modulator, on immobilized polysaccharide-based CSPs using SFC/UV and SFC/MS [78].

The increased availability of robust commercial instrumentation in recent years has coincided with an increased rate of application of SFC for chiral resolutions. New ways of performing SFC separations continue to emerge as advancements in column and instrument technologies enable a wider range of applications [79]. Besides, different strategies can be carried to scale-up [80] and improve productivity in preparative SFC, including stacked injections, larger feed, tandem combinations of columns, and the application of SFC–SMB, if larger amounts of enantiomers are necessary [26].

One recent example, illustrating the utility of tandem coupling columns strategy in SFC, described the stereoisomers resolution of a dihydropyridone derivative (a drug candidate for the treatment of type-2 diabetes) with multiple chiral centers [81]. In this specific case, the separation on a single column was insufficient; but tandem columns of different selectivity doubled the separation efficiency, increased resolution and effective separation of stereoisomers was achieved. Another efficient two-step method was developed for the SFC preparative separation of β-cypermethrin stereoisomers (synthetic pyrethroid insecticide) [82]. Firstly, β-cypermethrin was separated on a cellulose-derived CSP to afford two stereoisomeric pairs, and further resolved on an amylose-based CSP to obtain four enantiopure stereoisomers. The recoveries of four isomers ranged from 94.5 to 97.7%, and the enantiomeric excess values ranged from 92.19 to 97.53.

The evaluation of higher than typical linear velocities for SFC purifications on the preparative scale was also recently explored [83]. In many cases, running at high linear velocities showed a significant time saving and decreased consumption of mobile phase solvent, while also lowering energy consumption. These benefits can afford a more productive and environmentally friendly overall purification process.

Recently, a novel walk-up open-access (OA) approach to enable chiral analytical method development and preparative separation of enantiomers in early discovery chemistry using SFC was reported [84]. They proved that the key of the robust nature of the platform is the use of immobilized CSPs. After screening a diverse set of racemic drug candidates, they concluded that a simplified OA chiral SFC platform could successfully purify approximately 60% of the analyzed racemates. This streamlined OA workflow may enable medicinal chemists to rapidly purify enantiomers.

Nevertheless, SFC has several limitations compared to LC that cannot be overlooked, including lower flexibility from the viewpoint of separation adjustment based on the mobile phase. The solubility of polar compounds in SFC mobile phases is also limited. Reversed-phase mode is more difficult to implement in SFC due to the lack of polar supercritical fluid. Instrumentation in LC is cost effective and simpler than in SFC, and there is a huge knowledge available about LC and several scientific data available in literature. Even if nontoxic, massive carbon dioxide leak may create some problems to the operator in SFC remaining a constant risk [65]. Moreover, additional energy consumption for cooling or heating the carbon dioxide is needed [26].

### 2.3. Counter-Current Chromatography

In addition to LC and SFC, also counter-current chromatography (CCC) has been successfully applied, over the last few decades, in the field of chiral separation to obtain pure enantiomers [85,86]. This chromatographic technique has been widely used in the field of natural products chemistry [87,88]. It is based on liquid–liquid partition principle, which relies on the distribution of the target in an immiscible biphasic or multiphasic solvent system, being characterized by the absence of a solid support [89]. It is an excellent preparative separation tool due to its high load capacity, low solvent consumption, flexible elution modes, complete recoverability, continuous processing capability and easy scale-up [90,91,92]. In addition, CCC can be easily coupled with other online separation techniques [93]. All these features of CCC are very advantageous since the exploration of rapid and low-cost chiral separation technologies is of crucial importance. Nevertheless, in addition to these advantages, its main limitation is the relatively low separation efficiency of some chiral selectors, which requires a high enantioselectivity (separation factor (α) generally higher than 1.40) for the target racemate. In addition to the enantioselectivity, the chiral selectors also must have suitable solubility pattern and satisfactory phase distribution behavior [85]. β-Cyclodextrin derivatives are the most widely used chiral selectors in CCC considering its high chiral recognition ability [94].

The instrumental and technology advances, in addition to novel elution modes, such as gradient, dual-mode, multiple dual-mode, recycling, extrusion and concurrent elution, as well as to more specialized approaches, such as pH-zone-refining and biphasic chiral recognition technologies, allowed chiral separation by CCC to assume a more active role in the field of preparative chiral separation [95]. Especially since 2010, the development in this area has an accelerating growth with several recent publications found in literature [85,94,95], and currently may be assumed as a trend for preparative enantioseparation.

The most recent applications of CCC include a metal ion-improved complexation CCC, which was successfully developed for the enantioseparation of three dihydroflavones, (±)-hesperetin, (±)-naringenin and (±)-farrerol, compounds with very interesting and diverse biological/pharmacological activities [96]. Hydroxypropyl-β-cyclodextrin (HP-β-CD) was chosen as the chiral selector and Cu(II) ion as an additive to improve the enantioseparation efficiency of the dihydroflavones. The chiral recognition mechanism was investigated proving that the ternary complexes between (±)-hesperetin, HP-β-CD and ion were more stable when compared with the binary complexes of (±)-hesperetin and chiral selector.

A preparative enantioseparation of 2-(4-hydroxyphenyl)propionic acid (HPPA) (metabolite detected in multiple biofluids, such as urine and blood) by CCC using hydroxyethyl-β-cyclodextrin (HE-β-CD) as chiral selector was reported [97]. Two-phase solvent systems were selected and compared, composed of isobutyl acetate-aqueous phase (1:1, *v/v*) and *n*-butyl acetate-aqueous phase (1:1, *v*/*v*). Chiral selector concentration (HE-β-CD) of 0.1 mol L^−1^, pH value of 2.5, and temperature of 5 °C, were the best conditions. The recovery for HPPA enantiomers from CCC fractions was 80–90%, and the purities of both enantiomers were over 99.5%.

By using the same chiral selector (HE-β-CD), recycling CCC was successfully applied for the enantioseparation of 2-(4-bromomethylphenyl)propionic acid, a key synthetic intermediate for synthesis of loxoprofen, a nonsteroidal anti-inflammatory drug [98]. A biphasic solvent system comprising *n*-hexane/*n*-butyl acetate/0.1 mol/L citrate buffer solution with pH 2.4 (8:2:10, *v*/*v*/*v*) was selected. The concentration of HE-β-CD, pH of aqueous phase and temperature were optimized. Under optimum separation conditions, the recycling CCC afforded both enantiomers with enantiomeric excess of 98.0% and a recovery of 40.8–65.6%.

HE-β-CD chiral selector was also successfully applied for CCC preparative enantioseparation of 2-phenylbutyric acid (2-PBA) (antineoplastic agent) [99]. The typical factors affecting the enantioseparation efficiency were investigated and the two-phase solvent system consisting of *n*-hexane/butyl acetate/aqueous phase (7:3:10, *v*/*v*/*v*) was used. Under the optimized separation conditions, the purity was over 99.5% and the recovery reached 91–93%. In this study, a mathematical model was proposed, being in good agreement with the chromatographic results. Acetyltropic acid, an important synthetic intermediate for preparation of tropane alkaloid derivatives used as anticholinergic drugs and stimulants, was successfully enantioseparated by CCC using sulfobutyl ether-β-cyclodextrin, as the chiral selector, and a biphasic solvent system composed of *n*-butyl acetate/*n*-hexane/0.1 mol/L citrate buffer at pH = 2.2 containing 0.1 mol/L of sulfobutyl ether-β-cyclodextrin (7:3:10, *v*/*v*). Baseline separation was achieved for preparative enantioseparation of 50 mg of racemic acetyltropic acid [100].

Moreover, the preparative enantioseparation of synephrine (cardiovascular agent) by conventional and pH-zone-refining CCC achieved satisfactory results [101]. The enantioseparation by LC was also tried but with unsatisfactory results. The main factors that influence the efficiency of the CCC enantioseparation were investigated to optimize the separation conditions. Under optimum conditions, it was found that pH-zone-refining CCC was more efficient for the enantioseparation of synephrine.

### 2.4. Other Enantioseparation Methods

Other chromatographic techniques are described for the development of the enantioseparation method on a preparative scale, including gas chromatography (GC) [15]. Nevertheless, although various efforts and attempts have been made [102,103], this technique has still almost no relevance for preparative applications.

Membranes technologies are widely used in various separation processes due to their low cost, reliability, variety of materials, low energy consumption, eco-friendly and scalability compared with conventional separation processes. Based on their nature, the membranes can be classified into solid and liquid membranes [104]. Solid membranes are used extensively in different industries in processes like reverse osmosis, microfiltration and ultrafiltration. Liquid membranes consist of a liquid phase that acts as a membrane between two fluid phases (aqueous solutions or gas mixtures). The three basic types of liquid membranes are bulk liquid membrane (BLM), emulsion liquid membrane (ELM) and the supported liquid membrane (SLM) [105]. Many different types of chiral membranes and a variety of materials are described; however, their applications are still restricted to enantioseparation at a small scale, mainly, of pharmaceuticals and amino acids as exploratory projects [104,105,106,107]. Nevertheless, tremendous efforts have been made to develop chiral efficient membranes to achieve enantiomers with high enantiomeric purity [104,106].

As recent examples of application, new enantiopure lipophilic proton-ionizable crown ethers were synthesized as carriers for the active transport of protonated phenylethylamine, phenylglycinol, phenylalaninol, and ephedrine in BLM. Another example was the SLM enantioseparation of salbutamol (β2 agonist to treat bronchospasm) conducted in the hollow fiber SLM process [108].

Regarding solid membranes, enantioseparation of fluoxetine (SSRI antidepressant) has been reported by ultrafiltration with BSA, *Candida rugosa* lipase and α-cyclodextrin adsorbed on chiral selector-immobilized polyethersulfone membranes [109]. Homochiral metal–organic frameworks have also gained much attention because of their chiral properties and disposition for chiral separation [106]. A homochiral zeolitic imidazolate framework-8 (ZIF-8) membrane was synthesized by incorporating a natural amino acid, l-histidine, and presented selectivity for the *R*-enantiomer of 1-phenylethanol over the *S*-enantiomer, with enantiomeric excess up to 76% [110]. Taking inspiration from biological channels, a β-barrel transmembrane protein ferric hydroxamate uptake component A (FhuA) was re-engineered into a chiral channel protein (FhuAF4) to separate the racemate of D-/L-arginine with an enantioselectivity of 1.92 and an enantiomeric excess percentage of 23.91 [111]. Various methods to develop specific membranes by introducing chiral moiety into the polymeric membranes have been reported [23,112,113]. Recently, selective permeation of L-tyrosine through a functionalized single-walled carbon nanotube thin film nanocomposite membrane using D-tryptophan as the chiral probe achieved an enantiomeric excess up to 98.86% [114].

Despite the diversity of examples proving the feasibility for enantiomeric separation in preparative scale, so far, applications in drug discovery and development have not been sufficiently taken into account. Thus, more research in membranes development and applications are required.

## 3. Conclusions

Bearing in mind chiral molecules, enantioselective chromatography has become an indispensable tool in all the steps of drug development, especially in early stages, and some conclusions can be drawn considering the Medicinal Chemistry point of view leading with a large pleiad of chiral drugs. The demand for faster analytical and preparative enantioresolution techniques for bioactive compounds promoted the search for more efficient methods.

LC has turned out to be the technique of choice both during the drug discovery process, to achieve a few milligrams, or in scale-up during the clinical trial phases, needing kilograms of material. Nevertheless, over the last years, other enantioseparation methodologies, such as SFC and CCC, have been boosted and tend to became competitive methodologies to obtain enantiomerically pure compounds. The use of membranes is still restricted to a few pharmaceuticals, and need more investigation, but is pointed out as a promising methodology for further enantioseparation in the preparative scale due to its relative low cost and easy operation.
